# Acquired Comorbidities in Adults with Congenital Heart Disease: An Analysis of the German National Register for Congenital Heart Defects

**DOI:** 10.3390/jcm10020314

**Published:** 2021-01-16

**Authors:** Susanne J. Maurer, Ulrike M. M. Bauer, Helmut Baumgartner, Anselm Uebing, Claudia Walther, Oktay Tutarel

**Affiliations:** 1 Department of Electrophysiology, German Heart Centre Munich, TUM School of Medicine—Technical University of Munich, 80636 Munich, Germany; susanne.maurer@tum.de; 2 National Register for Congenital Heart Defects, Competence Network for Congenital Heart Defects, 13353 Berlin, Germany; ubauer@kompetenznetz-ahf.de; 3 Department of Cardiology III—Adult Congenital and Valvular Heart Disease, University Hospital Muenster, Albert-Schweitzer Campus 1, 48149 Muenster, Germany; Helmut.Baumgartner@ukmuenster.de; 4 Department of Congenital Heart Disease and Pediatric Cardiology, University Hospital Schleswig-Holstein, 24105 Kiel, Germany; anselm.uebing@uksh.de; 5 Department of Cardiology, University of Frankfurt, 60590 Frankfurt am Main, Germany; Claudia.Walther@kgu.de; 6 Department of Congenital Heart Disease and Paediatric Cardiology, German Heart Centre Munich, TUM School of Medicine—Technical University of Munich, 80636 Munich, Germany; 7 DZHK (German Centre for Cardiovascular Research), Partner Site Munich Heart Alliance, 80992 Munich, Germany

**Keywords:** adult congenital heart disease, comorbidities, cardiovascular risk factors

## Abstract

Background: As adults with congenital heart disease (ACHD) are getting older, acquired comorbidities play an important role in morbidity and mortality. Data regarding their prevalence in ACHD that are representative on a population level are not available. Methods: The German National Register for Congenital Heart Defects was screened for ACHD. Underlying congenital heart disease (CHD), patient demographics, previous interventional/surgical interventions, and comorbidities were retrieved. Patients <40 years of age were compared to those ≥40 years. Results: A total of 4673 patients (mean age 33.6 ± 10.7 years, female 47.7%) was included. At least one comorbidity was present in 2882 patients (61.7%) altogether, and in 56.8% of patients below vs. 77.7% of patients over 40 years of age (*p* < 0.001). Number of comorbidities was higher in patients ≥40 years (2.1 ± 2.1) than in patients <40 years (1.2 ± 1.5, *p* < 0.001). On multivariable regression analysis, age and CHD complexity were significantly associated with the presence and number of comorbidities. Conclusions: At least one acquired comorbidity is present in approximately two-thirds of ACHD. Age and complexity of the CHD are significantly associated with the presence of comorbidities. These findings highlight the importance of addressing comorbidities in ACHD care to achieve optimal long-term outcomes.

## 1. Introduction

The number of adults with congenital heart disease (ACHD) is increasing [1]. Currently, it is estimated that adults account for two-thirds of patients with severe and other forms of congenital heart disease (CHD) in the general population [2]. These ACHD patients have to deal with residua and sequelae related to their CHD as well as acquired comorbidities, especially as they are aging [3]. These comorbidities are not just innocent bystanders but can determine the outcome of ACHD patients [4,5,6,7]. Representative data regarding the burden of comorbidities among ACHD patients are missing. Single-center studies reporting comorbidity rates in this population have been published [8]. These are mainly from tertiary referral centers and are limited by a small number of patients involved with the additional caveat of referral bias. Data from inpatient databases are available as well [9], but only include the sickest patients and therefore, provide only biased estimates of population-level comorbidity prevalence [3]. A population-based study from the UK included a large number of patients below the age of 18 years, making assumptions for ACHD patients difficult [10]. Recently, Agarwal et al. used data from a commercial claims database representing the claims of employees and dependents on a large employer health benefit program in the United States (US) [3]. However, as the authors of this study stated themselves, there might be a selection bias with such an approach, because individuals included had commercial insurance, and therefore are expected to be generally healthier and of a higher socioeconomic status [3]. Additionally, such an approach does not represent the general population of the US, because it excludes 51% of the general population with other types of insurance or no insurance at all [3].

Therefore, the aim of this study is to provide data regarding the prevalence of acquired comorbidities in ACHD patients that are representative on a population level.

## 2. Material and Methods

The German National Register for Congenital Heart Defects provides a nationwide database and is representative for CHD in Germany [11]. Its data are not primarily gathered by tertiary referral centers but rather represent a community-based population (see also Supplementary File S1). The main cardiac diagnosis, all concurrent cardiac anomalies, as well as all performed cardiac catheter and surgical interventions are recorded in a database using the International Pediatric and Congenital Cardiac Code (IPCCC) published by the International Society for Nomenclature of Pediatric and Congenital Heart Disease (ISNPCHD; http://www.ipccc.net). In addition, extracardiac diagnoses and acquired diseases are recorded using the ICD-10 code (International Statistical Classification of Diseases and Related Health Problems) published by the World Health Organization (WHO; http://www.who.int/classifications/icd/en/). This information is extracted from medical reports by dedicated and trained staff.

The register was systematically screened in September 2017 for patients ≥18 years of age starting in the year 2012 with a medical report present. The medical reports of eligible patients were last updated in March 2019. At the time of screening, 50,837 patients were included in the register, and out of these, 20,562 patients were ≥18 years of age.

In addition to the underlying CHD, patient demographics and previous catheter interventional or surgical procedures were included in the analysis. Surgeries for cardiac pacemakers, internal cardiac defibrillators (ICD), and cardiac resynchronization devices (CRT) were registered separately. Diagnostic catheterizations were not included. Complexity of CHD was classified according to the Bethesda classification [12], which aims to group CHD in complex, moderate, and mild lesions. Mild lesions, for example, include isolated small ventricular septal defects, while Tetralogy of Fallot belongs to the group of CHD with moderate severity. CHD with great complexity include Eisenmenger syndrome, cyanotic CHD, and transposition of the great arteries, to name a few. Cardiac comorbidities like heart failure or arrhythmias, which can be sequelae of the CHD, were excluded, while coronary artery disease was included. Comorbidities were grouped using the ICD-10 code (Table A1, Appendix A).

Patients younger than 40 years of age were compared to those older than 40 years. Additionally, patients with a single-ventricle physiology (SVP) were compared to those with a biventricular CHD.

### 2.1. Ethics and Patient Involvement

The National Register was approved by the relevant Ethics Committee, and patients provided written informed consent for inclusion in the National Register and subsequent data use. Patients and public are continuously involved in all research projects, including design, conduct, and dissemination of the research from the National Register.

### 2.2. Data Availability Statement

The data underlying this article cannot be shared publicly due to data privacy reasons and the according German regulations.

### 2.3. Statistical Analysis

Statistical analyses were performed using SPSS version 25 (IBM Corp., Armonk, NY, USA) and MedCalc Statistical Software version 19.2.1 (MedCalc Software Ltd., Ostend, Belgium). Continuous variables are presented as mean ± standard deviation or median (interquartile range) depending on data distribution, whereas categorical variables are presented as number (percentage). Comparisons between groups were made using the Mann–Whitney U test or Student’s *t*-test or Fisher’s exact test or Chi-square test as appropriate. The relationship of age, complexity of heart disease (excluding those patients who could not be classified according to the Bethesda criteria [12]), and sex with the presence of comorbidities was assessed using multivariable logistic regression models. The relationship with number of comorbidities was assessed using multivariable negative binominal regression models. Multivariable negative binominal regression models were used instead of Poisson regression models because the number of comorbidities was overdispersed (i.e., conditional variance exceeded conditional mean). All tests were performed two-sided, and for all analyses, a *p*-value < 0.05 was considered statistically significant.

## 3. Results

Overall, out of 50,837 CHD patients in the National Register at the time of database query, we identified 4673 patients (mean age 33.6 ± 10.7 years, 47.7% female) meeting the inclusion criteria. A mild defect was present in 1369 patients (29.3%), a defect of moderate complexity in 1856 (39.7%), and a severe defect in 1323 (28.3%). In 125 patients (2.7%), the CHD could not be classified according to the Bethesda criteria. More detailed information is presented in Table 1.

### 3.1. Comorbidities

At least one comorbidity was present in 2882 patients (61.7%). According to the complexity of the CHD, at least one comorbidity was present in 55.3% of patients with a simple CHD, in 64.3% with a moderate CHD, and 63.7% with a complex CHD (*p* < 0.001). Female patients were as likely to have at least one comorbidity as male patients were (62.9% vs. 60.5%, *p* = 0.093). The most common comorbidities were endocrine and metabolic diseases (30.4%), circulatory system disorders (28.2%), and diseases of the nervous system (11.5%) (Table 2).

There was a statistically significant but weak correlation between the number of comorbidities and the number of cardiac surgeries (Spearman rank correlation coefficient 0.089, *p* < 0.0001) as well as the number of interventional catheterizations (Spearman rank correlation coefficient 0.079, *p* < 0.0001).

Coarctation of the aorta was the main CHD in 618 patients (13.2%). Hypertension was more common in this group compared to the other patients (48% vs. 10.8%, *p* < 0.001). This was not the case for ischemic heart disease (1.5% vs. 1.0%, *p* = 0.35).

### 3.2. Patients <40 Years of Age vs. Patients ≥40 Years

**Figure 1:**
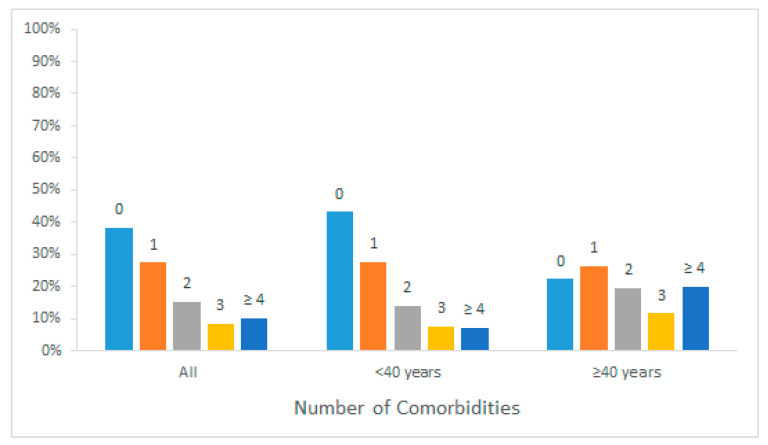
Number of comorbidities according to age group.

Altogether, 3584 patients (76.7%) were below the age of 40 years, while 1089 patients (23.3%) were 40 or older. There were more male (53.4%) than female (46.6%) patients in the age group <40 years, while there were more female (51.3%) than male (48.7%) patients in the age group ≥40 years (*p* < 0.01). Severe CHD were more common in those below the age of 40 years (Table 1). At least one comorbidity was present in 56.8% (*n* = 2036) of patients below 40 years of age and in 77.7% (*n* = 846) of patients over 40 years (*p* < 0.001). The number of comorbidities was also higher in patients ≥40 years (mean number of comorbidities 2.1 ± 2.1) than in patients < 40 years (1.2 ± 1.5, *p* < 0.001; Figure 1).

The most common acquired comorbidities in the age group <40 years were endocrine and metabolic diseases (24.3%), circulatory system disorders (21.5%), and diseases of the nervous system (10.4%). In the age group ≥40 years, diseases of the circulatory system were present in 549 patients (50.4%), endocrine and metabolic diseases in 547 (50.2%), and diseases of the nervous system in 166 (15.2%). Cardiovascular risk factors like arterial hypertension, diabetes mellitus, and hyperlipidemia were more common in the older age group (Table 2).

### 3.3. Single-Ventricle Physiology vs. Biventricular CHD

Out of the 4673 patients, 347 (7.4%) had a SVP. At least one comorbidity was present in 216 patients (62.2%) with SVP and in 2666 (61.6%) with a biventricular CHD (*p* = 0.82). Mean number of comorbidities was higher in SVP patients vs. biventricular CHD (1.7 ± 2.2 vs. 1.4 ± 1.7, *p* = 0.032).

### 3.4. Regression Analysis

On multivariable logistic regression analysis, age and complexity of CHD were significantly associated with the presence of at least one comorbidity (Table 3).

Furthermore, age and complexity as well as SVP were significantly associated with the number of comorbidities (Table 4).

## 4. Discussion

In the current study, at least one acquired comorbidity is present in more than 60% of ACHD patients from the German National Register for Congenital Heart Defects. Age and complexity of the CHD are significantly associated with the presence of at least one comorbidity, as well as with the number of comorbidities. Additionally, SVP is associated with the number of comorbidities.

In a recent study based on data from a commercial database representing the claims of employees and dependents of a large employer health benefit program in the US, around 30% of ACHD patients between 18 and 40 years of age, and 58% of those older than 40 years, had a comorbidity [3]. These numbers are lower than those in our study, in which at least one comorbidity was present in 57% of patients below 40 years of age and in 78% of patients over 40 years. A reason for these differences could be that the patients in the US study were commercially insured. As the authors pointed out, these individuals are generally healthier and have a higher socioeconomic status than the general US population [3]. Therefore, the study by Agarwal et al. [3] might have underestimated the prevalence of comorbidities in ACHD patients by excluding sicker patients with a probably lower socioeconomic status. In this study, ACHD patients had a 1.73- and 1.47-fold higher risk for non-CHD associated cardiovascular and non-cardiovascular comorbidities, respectively, than controls without CHD [3]. In contrast, the current study is based in Germany, which has a universally accessible healthcare system. Therefore, the study population might be more representative of the general population than that studied by Agarwal et al., as well as those from several other studies [9,10]. Singh et al. studied extra-cardiac comorbidities in ACHD patients using the US National Inpatient Sample database [9]. While the number of patients without any comorbidity was not reported in this study, a high prevalence of different comorbidities was present in their study cohort [9]. Unfortunately, a comparison with our cohort is difficult. First, their population was much older (mean age 56.9 years) compared to our cohort (mean age 33.6 years) and also to the cohort of Agarwal et al. (mean age 36.8 years). Furthermore, it included only ACHD patients who were hospitalized and therefore, it might be biased by only including sicker patients who needed inpatient treatment [3,9]. A study from the UK that assessed comorbidities in CHD patients used a primary care database [10]. This approach has the advantage that the study cohort is derived from a large and nationally representative study population [10]. However, 46.9% of patients included in this study were below the age of 19 years, and the prevalence of comorbidities was reported only for the whole cohort [10]. Therefore, pediatric and adult cases were mixed up. Additionally, only selected comorbidities were reported. Therefore, the burden of comorbidities in ACHD patients might be underestimated in this study. Our study has the strengths of including all comorbidities reported in a large sample of only adult patients derived from a large nationwide database which is representative for CHD in Germany [11].

In the current study, most common acquired comorbidities were endocrine and metabolic diseases (30.4%), circulatory system disorders (28.2%), and diseases of the nervous system (11.5%). In the older age group (>40 years), the prevalence of most comorbidities increased, and circulatory disorders like hypertension, ischemic heart disease, and stroke became more important. Compared to the prevalence in the German general population, significant comorbidities like stroke and renal disease were much more common in our study cohort [13,14]. For example, in the German Health Interview and Examination Survey for Adults, a large representative study of the German population, the stroke prevalence in the age group 40–49 years was 0.9% and for the age group 50–59 years it was 1.3% [14]. In contrast, it was 6.3% in our older age group, which had a mean age of 50 years. The higher incidence of stroke in ACHD patients has also been described in a Canadian study, in which the most important predictors of stroke were heart failure, diabetes mellitus, and a recent myocardial infarction [15]. Renal dysfunction is also an important predictor of outcome in ACHD patients, with a 3-fold higher mortality than normal in patients with moderate or severe renal dysfunction [4]. Cardiovascular risk factors like hypertensive disorders, diabetes mellitus, and hyperlipidemia contribute to the development of these comorbidities. These risk factors were present in a substantial number of patients in the current study, with approximately the same (diabetes mellitus) or even lower prevalence (hyperlipidemia) than in the general population [16,17]. In the younger age group (<40 years), hypertensive disorders were more common in our study compared to the general population, while their prevalence was similar to the general population in the older age group [18]. Our results are in accordance with the study by Agarwal et al., which reported a similar prevalence of hypertensive disorders and coronary artery disease as in our study, and an even higher prevalence for diabetes mellitus and hyperlipidemia compared to our cohort [3]. Another single-center study reported that even in a quite young cohort (median age 26 years) of ACHD patients, already 80% had at least one cardiovascular risk factor [19]. Accordingly, a Dutch study found an increased risk for coronary artery disease (CAD) in ACHD patients compared to the general population, with greater relative risk for those of younger age, women, and those with more severe CHD [20]. As ACHD patients are getting older, CAD becomes an important contributor to mortality [7]. Therefore, preventive measures are of the utmost importance, but unfortunately underutilized [21,22].

A limitation of this study is that comorbidities were retrieved from the diagnosis list in medical reports without access to laboratory data to assess the severity of the comorbidities like hyperlipidemia and endocrine disorders. Furthermore, outcome data like mortality were not available. Therefore, the impact of comorbidities on these outcome parameters could not be assessed. Additionally, the German National Register of Congenital Heart Defects only includes patients with CHD and no other groups of patients. Data on the German general population is unfortunately limited, because there is no general national register. We have tried to overcome this limitation by comparing our data to the data from other population-based German studies, which unfortunately used a different methodology, making comparison difficult. Therefore, we could not calculate standardized morbidity/mortality ratios.

## 5. Conclusions

In conclusion, at least one acquired comorbidity is present in the majority of ACHD patients from the German National Register for Congenital Heart Defects. Age, complexity of the CHD, and SVP are significantly associated with the presence of comorbidities. These findings highlight the importance of preventive measures as well as early diagnosis and therapy of comorbidities in ACHD care to achieve optimal long-term outcomes.

## Figures and Tables

**Table 1:** Baseline characteristics.

	All (*n* = 4673)	<40 Years (*n* = 3584)	≥40 Years (*n* = 1089)	*p*
Mean age (years ± SD)	33.6 ± 10.7	28.7 ± 5.2	49.9 ± 7.2	<0.001
Female	2228 (47.7%)	1669 (46.6%)	559 (51.3%)	<0.01
Bethesda classification of CHD				<0.001
Mild	1369 (29.3%)	1011 (28.2%)	358 (32.9%)	
Moderate	1856 (39.7%)	1386 (38.7%)	470 (43.2%)	
Severe	1323 (28.3%)	1091 (30.4%)	232 (21.3%)	
Other	125 (2.7%)	96 (2.7%)	29 (2.7%)	
Genetic syndrome	328 (7.0%)	300 (8.4%)	28 (2.6%)	<0.001
Comorbidities (≥1)	2882 (61.7%)	2036 (56.8%)	846 (77.7%)	<0.001
Cardiac surgery (≥1)	3841 (82.2%)	2988 (83.4%)	853 (78.3%)	<0.001
Device surgery (≥1)	579 (12.4%)	372 (10.4%)	207 (19.0%)	<0.001
Interventional catheterization (≥1)	1530 (32.7%)	1188 (33.1%)	342 (31.4%)	0.283
EP study/ablation (≥1)	397 (8.5%)	199 (5.6%)	198 (18.2%)	<0.001

SD, standard deviation; CHD, congenital heart disease; EP, electrophysiologic.

**Table 2:** Prevalence of comorbidities, *n* (%).

Comorbidities	All (*n* = 4673)	<40 Years (*n* = 3584)	≥40 Years (*n* = 1089)	*p*
Infectious diseases (all)	179 (3.8)	107 (3.0)	72 (6.6)	<0.001
Viral hepatitis	86 (1.8)	40 (1.1)	46 (4.2)	<0.001
Neoplasms (all)	102 (2.2)	54 (1.5)	48 (4.4)	<0.001
Malignant neoplasm	57 (1.2)	33 (0.9)	24 (2.2)	<0.01
Benign neoplasm	34 (0.7)	16 (0.4)	18 (1.7)	<0.001
Uncertain/unknown behavior	11 (0.2)	5 (0.1)	6 (0.6)	0.02
Diseases of the blood/blood-forming organs (all)	243 (5.2)	159 (4.4)	84 (7.7)	<0.001
Anemias	73 (1.6)	49 (1.4)	24 (2.2)	0.07
Endocrine and metabolic diseases (all)	1419 (30.4)	872 (24.3)	547 (50.2)	<0.001
Obesity	530 (11.3)	370 (10.3)	160 (14.7)	<0.001
Disorders of thyroid gland	422 (9.0)	284 (7.9)	138 (12.7)	<0.001
Type 1 diabetes mellitus	12 (0.3)	5 (0.1)	7 (0.6)	<0.01
Diabetes mellitus (excluding Type 1)	83 (1.8)	29 (0.8)	54 (5.0)	<0.001
Hyperlipidemia	144 (3.1)	39 (1.1)	105 (9.6)	<0.001
Mental and behavioral disorders	407 (8.7)	311 (8.7)	96 (8.8)	0.90
Diseases of the nervous system	539 (11.5)	373 (10.4)	166 (15.2)	<0.001
Diseases of the eye and adnexa	98 (2.1)	65 (1.8)	33 (3.0)	0.02
Diseases of the ear and mastoid process	72 (1.5)	49 (1.4)	23 (2.1)	0.09
Diseases of the circulatory system (all)	1318 (28.2)	769 (21.5)	549 (50.4)	<0.001
Hypertensive diseases	730 (15.6)	412 (11.5)	318 (29.2)	<0.001
Ischemic heart disease	51 (1.1)	22 (0.6)	29 (2.7)	<0.001
Cerebral infarction/stroke	161 (3.4)	92 (2.6)	69 (6.3)	<0.001
Atherosclerosis precerebral/cerebral arteries	13 (0.3)	5 (0.1)	6 (0.6)	0.02
Atherosclerosis	10 (0.2)	2 (0.1)	8 (0.7)	<0.001
Diseases of the respiratory system (all)	388 (8.3)	240 (6.7)	148 (13.6)	<0.001
COPD/asthma/chronic bronchitis	176 (3.8)	95 (2.7)	81 (7.4)	<0.001
Diseases of the digestive system	311 (6.7)	185 (5.2)	126 (11.6)	<0.001
Diseases of the skin	123 (2.6)	97 (2.7)	26 (2.4)	0.67
Diseases of the musculoskeletal system and connective tissue	344 (7.4)	221 (6.2)	123 (11.3)	<0.001
Diseases of the genitourinary system (all)	247 (5.3)	151 (4.2)	96 (8.8)	<0.001
Glomerular disease, renal tubulo-interstitial disease, renal failure	135 (2.9)	73 (2.0)	62 (5.7)	<0.001
Pregnancy, childbirth, and the puerperium	76 (1.6)	58 (1.6)	18 (1.7)	0.89
Conditions originating in the perinatal period	18 (0.4)	16 (0.4)	2 (0.2)	0.28
Congenital malformations, deformation, and chromosomal abnormalities	390 (8.3)	335 (9.3)	55 (5.1)	<0.001
Injury, poisoning	156 (3.3)	98 (2.7)	58 (5.3)	<0.001
External causes of morbidity and mortality	17 (0.4)	13 (0.4)	4 (0.4)	1.00

COPD, chronic obstructive pulmonary disease.

**Table 3:** Association between age, complexity of CHD, and gender with presence of at least one comorbidity.

	Univariate		Multivariate	
Variable	OR (95% CI)	*p*	OR (95% CI)	*p*
Age (in years)	1.05 (1.04–1.06)	<0.0001	1.05 (1.04–1.06)	<0.0001
Male	0.89 (0.79–1.01)	0.06		
Biventricular CHD	0.97 (0.78–1.22)	0.82		
Moderate CHD	1.46 (1.26–1.68)	<0.0001	1.53 (1.32–1.77)	<0.0001
Severe CHD	1.42 (1.22–1.66)	<0.0001	1.57 (1.34–1.84)	<0.0001

OR, odds ratio; CHD, congenital heart disease.

**Table 4:** Association between age, complexity of CHD, and gender with number of comorbidities.

	Incidence Rate Ratio	95% Confidence Interval	*p*
Age (in years)	1.030	1.026–1.033	<0.001
Male	0.983	0.909–1.062	0.658
Moderate CHD	1.245	1.132–1.370	<0.001
Severe CHD	1.351	1.210–1.509	<0.001
Biventricular CHD	0.793	0.677–0.928	0.004

CHD, congenital heart disease.

## Data Availability

The data underlying this article cannot be shared publicly due to data privacy reasons and the according German regulations.

## Supplementary Materials

The following are available online at https://www.mdpi.com/2077-0383/10/2/314/s1, File S1: German Study Group Competence Network for Congenital Heart Defects.

## Appendix A

**Table A1:** Comorbidities according to ICD-10.

Comorbidities	ICD-10 Codes
Infectious diseases	A00-B99
Viral hepatitis	B15-B19
Neoplasms	
Malignant neoplasm	C00-C97
In situ neoplasm	D00-D09
Benign neoplasm	D10-D36
Uncertain/unknown behavior neoplasm	D37-D48
Diseases of the blood/blood-forming organs	D65-D90
Anemias	D50-D64
Endocrine, nutritional, and metabolic diseases	E00-E90
Disorders of thyroid gland	E00-E07
Type 1 diabetes mellitus	E10
Diabetes mellitus (excluding Type 1)	E11-E14
Obesity	E66
Hyperlipidemia	E78.0-E78.5, E78.8-E78.9
Mental and behavioral disorders	F00-F99
Diseases of the nervous system	G00-G99
Diseases of the eye and adnexa	H00-H59
Diseases of the ear and mastoid process	H60-H95
Diseases of the circulatory system	I00-I99 excl. I27.28-I27.9, I30-I39, I42-I52, I71
Hypertensive diseases	I10-I15
Ischemic heart disease	I20-I25
Cerebral infarction/stroke	I63-I64
Atherosclerosis precerebral/cerebral arteries	I65-I66, I67.2
Atherosclerosis	I70
Diseases of the respiratory system	J00-J99
COPD/asthma/chronic bronchitis	J40-J47
Diseases of the digestive system	K00-K93
Diseases of the skin	L00-L99
Diseases of the musculoskeletal system and connective tissue	M00-M99
Diseases of the genitourinary system	N20-N99
Glomerular disease, renal tubulo-interstitial disease, renal failure	N00-N19
Pregnancy, childbirth, and the puerperium	O00-O99
Conditions originating in the perinatal period	P00-P96
Congenital malformations, deformation, and chromosomal abnormalities	Q00-Q99 excl. Q20-Q28
Injury, poisoning	S00-T98 excl. T80-T88
External causes of morbidity and mortality	V01-Y84

ICD-10, International Statistical Classification of Diseases and Related Health Problems.
